# Recombinant Keratinocyte Growth Factor 1 in Tobacco Potentially Promotes Wound Healing in Diabetic Rats

**DOI:** 10.1155/2014/579632

**Published:** 2014-03-24

**Authors:** Zhi-Guo Feng, Shi-Feng Pang, Ding-Jiong Guo, Yue-Tao Yang, Bin Liu, Ji-Wei Wang, Ke-Qin Zheng, Yi Lin

**Affiliations:** ^1^College of Life Science, Anhui Agricultural University, Hefei 230036, China; ^2^Department of Biology, Guangdong Medical College, Dongguang 523808, China; ^3^Department of General Surgery, Cixi People's Hospital, Ningbo 315300, China; ^4^Traumatic Medicine Center, Lishui People's Hospital, Lishui 315300, China

## Abstract

Keratinocyte growth factor 1 (KGF1) is a growth factor that promotes epidermal cell proliferation, migration, differentiation, and wound repair. It is expressed at low levels in a form of inclusion body in *E. coli.* In order to increase its expression and activity, we produced tobacco plants expressing KGF1 via *Agrobacterium-mediated* transformation using a *potato virus X (PVX)*-based vector (pgR107). The vector contained the sequence encoding the KGF1 gene fused with a green florescence protein. The recombinant plasmid was introduced into leaf cells of *Nicotiana benthamiana* (a wild Australian tobacco) via *Agrobacterium-mediated* agroinfiltration. As determined by fluorescence and Western blot of leaf extracts, the KGF1 gene was correctly translated into the tobacco plants. The recombinant KGF1 was purified from plant tissues by heparin affinity chromatography, and cell proliferation in NIH/3T3 cells was stimulated by the purified KGF1. The purified KGF1 was also applied to the wounds of type-II diabetic rats. KGF1 had accumulated to levels as high as 530 **μ**g/g fresh weight in the leaves of agroinfected plants. We show that plant-derived KGF1 can promote the proliferation of NIH/3T3 cells and have significant effects on the type-II diabetic rat. The present findings indicated that KGF1 from tobacco maintains its biological activity, implying prospective industrial production in a plant bioreactor.

## 1. Introduction

Keratinocyte growth factor (KGF1) is a member of the fibroblast growth factor family (also denoted as FGF7). KGF1 is produced by cells of mesenchymal origin and secreted via a paracrine secretion mechanism. It binds to epithelial cell-specific receptors and plays important roles in wound repair, embryonic development, tumor generation, and immunologic reconstitution [1–7]. KGF1 promotes cell proliferation and is expressed at high levels during wound repair. KGF1 can also promote epidermal cell proliferation, migration, and differentiation and can increase proliferation and differentiation of skin hair follicles and sebaceous gland precursor cells [6, 8–10]. Previously, we expressed KGF1 in* E. coli *and found that KGF1 expression levels were low and mainly confined to a form of inclusion body, which causes difficulties in KGF1 purification and limits the usefulness of KGF1 in basic and applied pharmacology and pathology researches. In this study, we employed a tobacco viral expression system to improve the expression efficiency and activity of KGF1.

Plants have been studied extensively for the production of pharmaceutical proteins, as they represent an inexpensive and scalable alternative to common expression systems. Plants offer advantages over microbial or mammalian host systems: the bioprocessing is more effective, they have all of the cellular machinery needed to complete posttranslational modifications of proteins, and they are intrinsically safe. The use of the* Potato virus X* (PVX) vector in such systems is also safe, as there is no evidence that PVX is transmitted by naturally occurring vectors, for example, insects, nematodes, and fungi. Moreover, the virus is not transmitted through seeds or pollen, so risk of its accidental release into the environment is extremely small. Therefore, environmentally safe conditions are attainable with only a moderate investment in infrastructure. The combination of a plant production system and a PVX-based vector is a promising platform for the production of recombinant proteins [11–15].

Distinct from stable transgenic plants, plant RNA viruses provide a temporary, transient expression system. These viruses are engineered to carry and replicate foreign genes in susceptible host plants. The sequences delivered by viruses into the infected plant cell remain part of the virus genomic RNA and do not integrate into the plant genome. Moreover, the foreign transcripts are amplified by the viral replicase in the cell cytoplasm and are not inherited. Plant viruses are plausible transient expression vectors since the introduced genes are expressed at high levels and the purification of the products from the plants is simple and inexpensive.

## 2. Methods

### 2.1. Vector Construction

Plasmids pgR107 and pgR107-smGFP and KGF1 DNA were generously provided by Dr. Xingzhi Wang (Institute of Genetics and Cytology, Northeast Normal University, Changchun, China). The parental plasmid pUCKGF1 contains a ClaI/SmaI/SalI multiple cloning site surrounded by two subgenomic viral coat protein (CP) promoters. The pgR107 plasmid contains a 35S promoter, which drives synthesis of infectious PVX transcripts in plants [16]. The KGF1 gene (Gene ID: 14178) was inserted into the cloning site of the PVX within pgR107, and its expression was controlled by one of the CP promoters. The* KGF1-GFP* sequence flanked by ClaI and SalI digestion sites was amplified by PCR and cloned into the pgR107 vector, resulting in pgR107-*KGF1-GFP*.

### 2.2. Plant Material and Inoculation Conditions

The transfection of pgR107-*KGF1-GFP* and pgR107-smGFP plasmids was conducted by a freeze-thaw method using* A. tumefaciens* GV3101 with* N. benthamiana* plants (4-5 weeks of age) at approximately the ten-leaf stage [17]. The recombinant agrobacteria were separately cultured in 50 mL LB (Luria-Bertani) medium (supplemented with kanamycin (50 mg/L), rifampicin (50 mg/L), tetracycline (12.5 mg/L), 10 mM MES, and 20 *μ*M acetosyringone) overnight at 28°C. The cells were collected by centrifugation at 1,500 g for 10 min and resuspended and incubated in liquid minimal medium agar without shaking (10 mM MgCl_2_, 10 mM MES, and 100 *μ*M acetosyringone) for 2-3 h at room temperature before infiltration. After agrobacterial infiltration onto the underside of the tobacco leaves, the leaves were placed in a dark humid atmosphere for 24 h to recover from the treatment. The recombinant protein expression was confirmed by green fluorescence illumination with an ultraviolet light as well as by TCS SP5 confocal laser-scanning microscope (Leica Microsystems CMS GmbH, Germany).

### 2.3. Protein Extraction and Purification

Seven days after infection, the* N. benthamiana* leaves were harvested and frozen in liquid nitrogen. The leaves were then ground into an extraction buffer (50 mM PBS, 1 mM PMSF, and pH 9.5), at a ratio of 3 mL per gram leaf material. Extracts were collected by centrifugation at 13,520 g for 20 min at 4°C.* KGF1-GFP* fusion protein was purified by heparin affinity chromatography and identified by sodium dodecyl sulfate polyacrylamide gel electrophoresis (SDS-PAGE). The fusion protein was further cleaved with bovine enterokinase (rbEK) to remove the end of the* C*-terminal peptide for obtaining active KGF-1 protein; KGF-1 was separated from smGFP by heparin affinity chromatography.

### 2.4. SDS-PAGE and Western Blot

For Western blot analysis, cellular lysates were transferred onto Immobilon polyvinylidene difluoride (PVDF) membranes (Perkin Elmer, Boston, MA, USA) and immunoblotted with a monoclonal mouse anti-KGF1 antibody (Abcam Inc., USA) (diluted to 1 : 1,500 in 5% DM/PBST) according to the manufacturer's instructions. Immunoreactive bands were visualized with Western blotting luminal reagents (Western Blotting Luminol Reagent sc-2048 Trial Kit, Santa Cruz Biotechnology Inc., CA, USA).

### 2.5. ELISA Assay of KGF1-GFP in* N. benthamiana* Plants

The powder of ground leaves (0.5 g fresh weight) was homogenized with 1.5 mL of 50 mM PBS (pH 9.5) and centrifuged for 20 min at 13,520 g to obtain total protein extracts from transfected and control plants. The concentration of total soluble protein was estimated by the Bradford assay method. For enzyme-linked immunosorbent assay (ELISA), serial dilutions of total protein extracts from 1 : 50 to 1 : 1,000 in phosphate-buffered saline (PBS) were incubated in 96-well polyvinyl chloride microtiter plates for 2 h at 37°C. The plates were then incubated with 1% fat-free dry milk (DM) for 1 h at 37°C. After the plates were washed three times with PBS containing 0.05% Tween 20 (PBST), the mouse anti-KGF1 antibodies (diluted to 1 : 2,000 in 1% DM/PBST) were added (50 *μ*L per well) to the testing wells and the plates were incubated for 2 h at 37°C. Next, a rabbit anti-mouse IgG-horseradish peroxidase conjugate (Sigma; 1 : 2,000 in 1% DM/PBST) was added to the testing wells and the plate was incubated for 1 h at 37°C. The plate was then developed with Slow TMB substrate (Pierce) for 15 min at 23°C, and the reaction was subsequently terminated by adding an equal volume of 0.5 M H_2_SO_4_. To construct the standard curve, bacteria-derived KGF1 was diluted with PBS to concentrations ranging from 0 to 10 *μ*g/mL and processed as above. All of the ELISA assays results are means of triplicated experiments.

### 2.6. NIH/3T3 Cell Proliferation Assay

For cell counting and the construction of growth curves, NIH/3T3 cells were cultured in Dulbecco's modified Eagle's medium (DMEM) supplemented with 10% fetal bovine serum. At 80% confluence, cells were collected and split. Cells (10^5^/mL) were seeded in 96-well plates (50 *μ*L/well) and incubated at 37°C, in a 5% CO_2_ atmosphere for 24 h. The media were replaced with keratinocyte serum-free medium (K-SFM, Sigma). Cells were divided into 4 groups with 6 replicates in each group. The control group received only K-SFM (free of KGF1), and the positive control group received medium containing 100 ng/mL commercial standard KGF-1. The experimental groups received media containing 100 ng/mL purified plant KGF1. Cells were incubated at 37°C in a 5% CO_2_ atmosphere for 24, 36, 48, 60, and 72 h, respectively. After the treatments, cells were collected by mild trypsinization and counted for cell numbers to construct a growth curve. Cell morphology was monitored at 72 h.

### 2.7. Animal Model for Wound Healing

We took care of all animals according to the Guiding Principles in the Care and Use of Animals. Female Sprague-Dawley rats (~200 g) were subjected for induction of type-II diabetes by a single intraperitoneal injection of streptozotocin (STZ, Sigma; 30 mg/kg in citrate buffer). Diabetic rats were injected intraperitoneally with 8% chloral hydrate as an anaesthetic and then were fixed on a simple platform to facilitate cutting of hair and disinfection of the back skin with iodophor. Each rat was given two wounds (2 cm in diameter) by application of weights, heated to 95°C in a hot water bath, to its back bilateral to the spinal axis. Plant KGF-1 (500 ng/mL in saline) was applied to the right wound, and an identical amount of saline was applied on the left as in the control group. Application of 500 ng/mL of standard KGF1 served as the positive control. The administration method consisted of applying either 200 *μ*L KGF-1 solution or saline once every other day for two weeks. Photographic records were obtained throughout the experiments, for a total period of 28 d.

### 2.8. Pathology Evaluation

Rat skin tissue samples were collected from three groups: plant KGF1, positive control, and control. Samples were embedded in paraffin, sliced, subjected to Masson's staining, and examined with light microscopy.

### 2.9. Statistical Analysis

The data were expressed as the mean ± SEM. Statistical significance was determined by Student's *t*-test when there were two experimental groups. For more than two groups, statistical evaluation of the data was performed using one-way analysis of variance (ANOVA), followed by Dunnett's post hoc test, with *P* < 0.05 considered significant.

## 3. Results

### 3.1. Plasmid Construction

**Figure 1:**
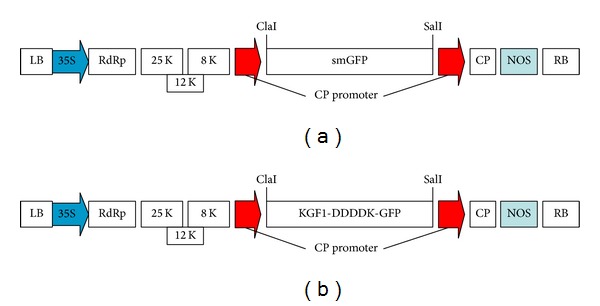
(a) Schematic map of plasmid pgR107-smGFP. (b) Schematic map of plasmid pgR107-KGF1-GFP. LB and RB, left and right border sequences, respectively, of T-DNA of* A. tumefaciens*; NOS, transcriptional terminator of nopaline synthase gene of* A. tumefaciens*; RdRp, viral RNA-dependent RNA polymerase; K, kDa; 25 K, 12 K, and 8 K, viral movement proteins; CP, viral coat protein; 35S, cauliflower mosaic virus 35S promoter.

We constructed binary virus vectors pgR107-*KGF1-GFP*. The DNA coding sequences for* KGF1-GFP* were controlled by a strong subgenomic promoter of the PVX coat protein. Therefore, the genes could be expressed rapidly in* N. benthamiana* concurrently with viral replication. To increase the expression of KGF1, we inserted the Kozak sequence at the 5′ end of KGF1 and changed some of the native KGF1 sequence codons to plant preferred codons without altering the amino acid composition of the protein. Furthermore, the DDDDK enterokinase cleavage site was inserted into the* KGF1-GFP* gene (Figure 1(b)), which allowed complete removal of the smGFP portion from* KGF1-GFP* via a cleavage reaction.

### 3.2. Agroinfection of* N. benthamiana* Plants

**Figure 2:**
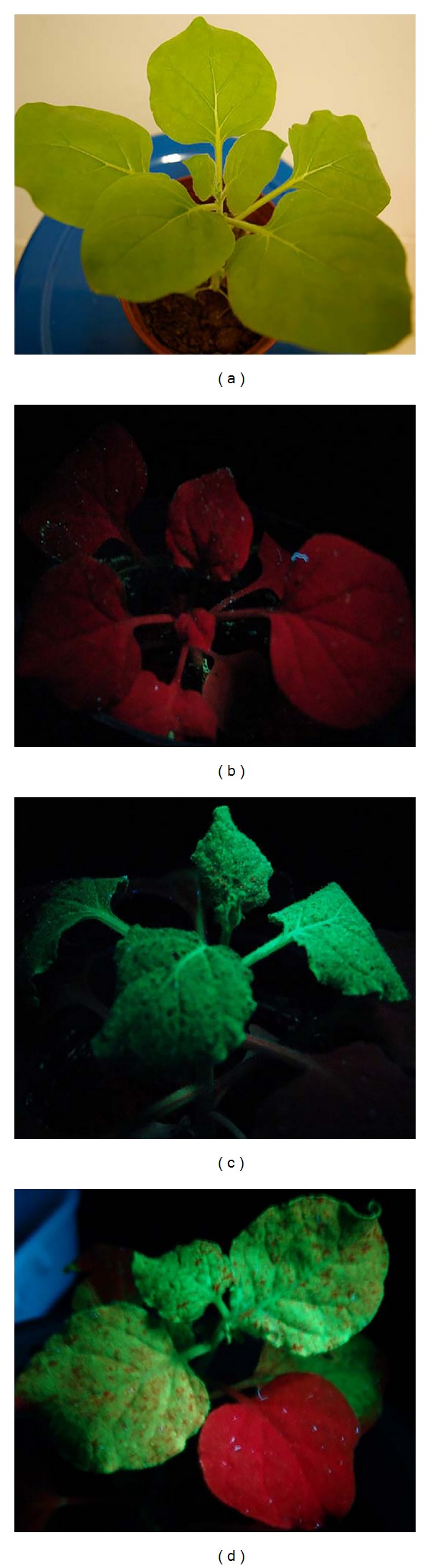
Green fluorescent images produced by UV illumination. (a) Control plants (noninfected) under visible light. (b) Noninfected plant under UV illumination. (c) Expressed KGF1-GFP visualized at 7 dpi under UV illumination. (d) Expressed smGFP visualized at 7 dpi under UV illumination.

**Figure 3:**
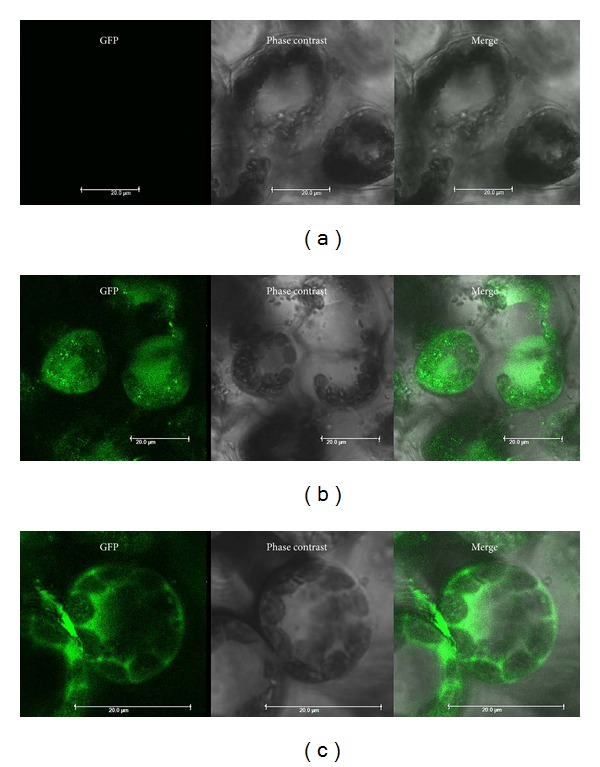
Confocal microscopy images. (a) Noninfected control tobacco cells. (b) KGF1-GFP fusion gene expression in tobacco cells. (c) smGFP expression in tobacco cells. Bar = 20 *μ*m.

The* N. benthamiana* plants agroinfected with* A. tumefaciens* containing pgR107-smGFP (Figure 1(a)) and pgR107-*KGF1-GFP* (Figure 1(b)) developed the characteristic mosaic symptoms of PVX infection. Expression of the recombinant protein was visualized by fluorescence in plants agroinfected with GV3101/pgR107-*KGF1-GFP*. Under ultraviolet illumination, green fluorescent spots were clearly visible on agroinfected leaves of* N. benthamiana* at 3-4 days postagroinfection (dpi). Diffuse green fluorescence of smGFP and* KGF1-GFP* was visible in stems and leaves above the infected leaf on the day after agroinfection. The highest accumulation of smGFP (Figure 2(d)) or* KGF1-GFP* (Figure 2(c)) was in leaves and stems at approximately 7 dpi, after which the amounts of smGFP and* KGF1-GFP* tended to decrease. Confocal microscopy analysis revealed that the* KGF1-GFP* and smGFP proteins were localized in the cytoplasm (Figure 3). These results demonstrate that agroinfection is an efficient method of initiating PVX infection and that the PVX-based binary vector is an efficient transient expression vector for production of* KGF1-GFP* in* N. benthamiana*.

### 3.3. SDS PAGE and Western Blot

**Figure 4:**
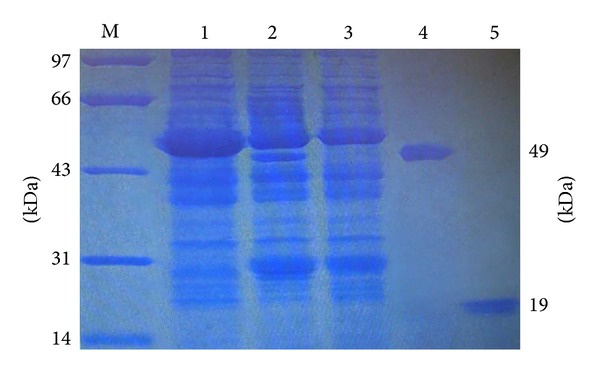
SDS-PAGE analysis. M: molecular weight standard. 1: WT noninoculated control tobacco leaves. 2: pgR107-KGF1-GFP. 3: pgR107. 4: KGF1-GFP (49 KDa) purified from tobacco leaves at 7 dpi. 5: KGF1 (19 KDa) after cleavage and separation from KGF1-GFP fusion protein.

**Figure 5:**
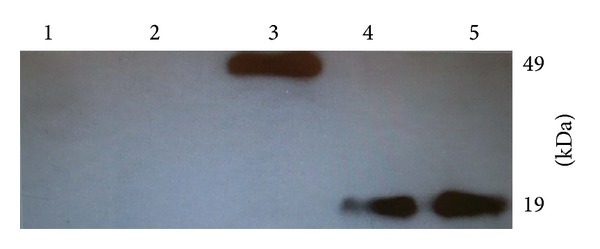
Western blot of purified KGF1-GFP and KGF1. KGF1-GFP and KGF1 from tobacco leaves were resolved by SDS-PAGE, blotted, and probed with mouse anti-KGF1 antibody. 1: noninoculated control tobacco leaves. 2: agroinfected with GV3101/pgR107. 3: agroinfected with GV3101/pgR107-KGF1-GFP(49 KDa). 4: KGF1 (19 KDa) after cleavage and separation from KGF1-GFP fusion protein. 5:* E. coli* recombinant KGF1.

The analysis of KGF1-GFP (49 kDa) expression by SDS-PAGE showed that* KGF1-GFP* (Figure 4) was expressed at high levels. The KGF1 protein was cleaved from the* KGF1-GFP* fusion protein by bovine enterokinase.* KGF1-GFP* and KGF1 (Figure 4) were purified from the cellular lysates of* N. benthamiana* leaves. Western blots of proteins purified from* N. benthamiana* leaves further confirmed the expression of* KGF1-GFP* fusion protein and KGF1. The results showed that* KGF1-GFP* was expressed (Figure 5).

To reliably quantify the level of expression, leaf extracts were analyzed by direct ELISA. The ELISA results showed that* KGF1-GFP* was expressed at high levels in the cytosol, accumulating to approximately 530 *μ*g per gram fresh leaf weight. The stability of the* KGF1-GFP* fusion protein may well protect its KGF1 portion from degradation by hydrolase. Native KGF1 can be obtained after cleavage of the GFP. In other systems using plant virus-mediated transient expression, the reported yields of recombinant pharmaceutical protein purified from tobacco leaves ranged from 0.00003 to 2 mg/g leaf fresh weight.

### 3.4. NIH/3T3 Cell Proliferation Assay Treated by KGF1

**Figure 6:**
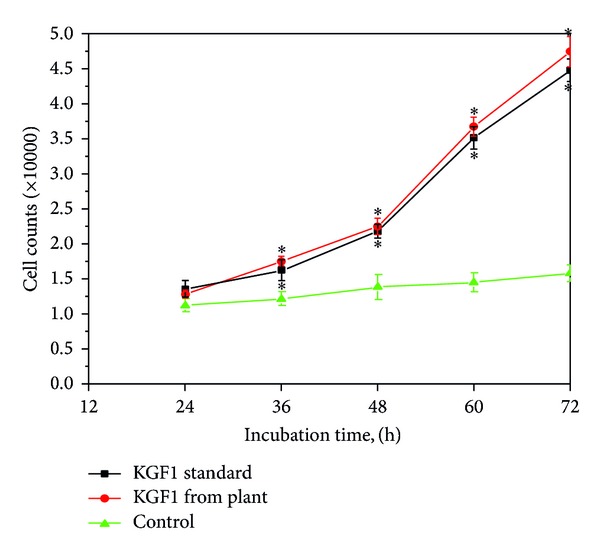
Growth curve of NIH/3T3 cells. KGF1 standard protein and plant-derived KGF1 protein have the same effect on proliferation of NIH/3T3 cell. Both promote faster cell proliferation than control (K-SFM). Values are mean ± SD of three independent experiments. *P < 0.05 versus control group.

We used K-SFM as the control and KGF1 standard protein as the positive control. Cell count results show that the KGF-1 standard and plant-derived KGF1 promote significantly more NIH/3T3 cell proliferation than that observed in the control group, especially in the time period from 36 h to 72 h after treatment (Figure 6). The proliferation effect of KGF-1 standard and plant-derived KGF1 seems to be of no difference. After 72 h, both two kinds of KGF-1 stimulated cell proliferate for 3-fold more than the cell number in 24 h. These results confirm that the plant-derived KGF1 protein can promote cell proliferation. When compared with the standard KGF1 protein from bacteria, there is no significant difference.

### 3.5. Wound Healing Evaluation Assay

**Figure 7:**
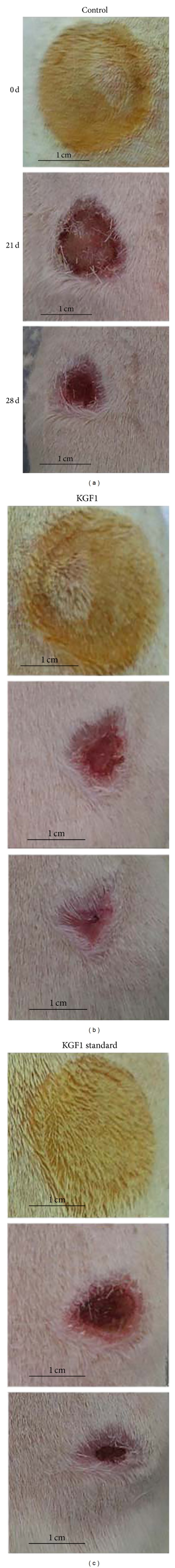
Wound healing assay. Left column: normal saline control group; center column: plant KGF1 group; right column: KGF1 standard protein group.

In order to evaluate the wound healing activity of plant-derived KGF1, we performed a healing evaluation assay in SD rats. Results are shown in Figure 7. We used 0.9% normal saline solution as the control. It was seen that the wounds treated by KGF-1 and KGF-1 standard recovered much more quickly with better skin appearance (Figure 7). After the 28th day, the wounds treated with plant KGF-1 showed wound closured mostly, while the wounds in the control had obvious wound surface. The healing time of the control group was 35.80 ± 0.37 days, while that of the plant-derived KGF1 group was 28.19 ± 0.62 days and that of the KGF1 standard protein group was 28.84 ± 0.77 days. These results show that the wound healing time of the plant-derived KGF1 group was significantly shorter than that of the control group, while there was no significant difference between the plant-derived KGF1 group and the KGF1 standard control group.

### 3.6. Masson's Staining

**Figure 8:**
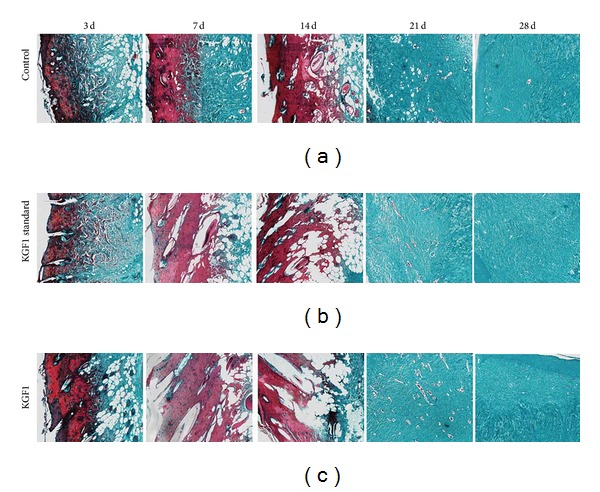
Masson's staining assay imaging. Top row: normal saline control group; center row: KGF-1 standard protein group; bottom row: plant-derived KGF-1 group. Magnification: 200x.

Masson's staining stained the collagen fibers of the tissue green. After 3 days, we observed that the rat skin epithelia were deeply stained in all groups.  Figure 8 shows increased signs of necrosis, thickening layers, formation of blood crust on the surface, and dermal fiber degeneration. Particularly, KGF1 groups (the KGF1 standard group and recombinant KGF1 group) showed more serious dermis denaturing accompanied by vacuolar degeneration. At the 7th and 14th days, the KGF1 groups showed severe edema, more profound necrosis, severe structural damage, increased necrotic degeneration, and pyknosis more than the control group. In the meantime, the necrosis organization also began to be cleared, and this is conducive to the remodeling of latter tissue. On the 21st day, tissue remodeling had been completed, and collagen formation was also observed. The administration groups' skin collagen was better arranged than that of the control group. Also, much more neovascularization was observed in the administration groups, which is favorable for supplying nutrients to the tissue. By the 28th day, the tissue had completed epithelization. The collagen fibers in the plant-derived KGF1 treated group and KGF-1 standard protein treated group were in good arrangement. On the contrary, the control group displayed disordered collagen.

## 4. Discussion

Here, we report an expression system using* Nicotiana benthamiana* to produce KGF-1.* N. benthamiana* leaves were infiltrated with* Agrobacterium tumefaciens* strains containing pgR107 binary vectors that were designed to combine the advantages of* A. tumefaciens*-mediated transfection and viral infection. We inserted the coding sequence of KGF-1 and smGFP into the cloning sites of the viral binary vector to construct the experimental plasmids pgR107-KGF1-GFP. Viral replication and expression of the recombinant fusion gene occurred concurrently in agroinfiltrated* N. benthamiana.*

A strong subgenomic promoter of the PVX coat protein controlled the DNA coding sequences for* KGF1-GFP*. This assures that the genes will be expressed rapidly in* N. benthamiana* concurrently with viral replication. Also, the Kozak sequence at the 5′ end of KGF1 is useful for increasing the expression of KGF1. Furthermore, alternating some of the native KGF1 sequence DNA codons to plant-preferred codons without changing the amino acid composition of the protein may increase the expression as well.

KGF1 is an epithelial cell-specific growth factor that promotes epidermal cell proliferation, migration, and differentiation, skin wound healing, and improvement of the quality of wound healing. Wound base fibroblasts are able to synthesize and release KGF1, which induces epidermal cell proliferation and migration toward the wound.

Traditional methods of KGF1 production involve animal tissue extraction and prokaryotic expression in* E. coli*. The first method, however, suffers from low yield and is expensive. The second method has the problem of unstable expression in* E. coli*, mainly because the exogenous protein is expressed in inclusion bodies and so requires renaturation of the inclusion body protein. The biological activity of this product is very low. We expressed KGF1 in tobacco by using* Potato virus X* (pGR107), thereby seeking a low cost, simple method with a high yield of KGF1. In the present study, we constructed a plant viral expression vector pGR107-*KGF1-GFP* and then transiently expressed this construct in tobacco. We observed clear green fluorescence in infected tobacco leaves by ultraviolet excitation. Laser confocal microscopy scanning of the tobacco leaf cells revealed expression of the fusion protein containing KGF1. These pieces of data confirm that the fusion protein can be highly expressed in the tobacco leaf and can be excited to produce green fluorescence. After grinding the leaf and purifying the fusion protein, we validated that the* KGF1-GFP* fusion protein was successfully expressed in tobacco by SDS-PAGE and Western blot.

NIH/3T3 cell proliferation assay results show that plant-derived KGF1 can significantly promote the proliferation of NIH/3T3 cells, with the same activity of the KGF1 standard protein. Studies have shown that KGF-1 has a therapeutic effect on diabetic ulcers [18, 19]. In the present study, we used plant-derived KGF1 in the treatment of wounds in diabetic rats. The results show that the plant-derived fusion protein* KGF1-GFP*, after cleavage by erepsin, yields KGF1 that can significantly accelerate the speed of wound healing of diabetic rats compared with rats treated with normal saline. Masson's staining shows that the plant-derived KGF1 has the same effects as KGF-1 standard protein, significantly increasing wound epithelialization and promoting necrotic tissue clearance and collagen synthesis compared with normal saline. These results indicate that KGF1 has positive effects on the treatment of difficult type-II diabetic ulcers, and the results also provide a theoretical basis for the application of KGF-1 in clinical type-II diabetes therapy.

## 5. Conclusion

In conclusion, the evidence demonstrated that tobacco plants expressed KGF1 via* Agrobacterium*-mediated transformation using a* Potato virus X*- (PVX-) based vector (pgR107). The plant-derived KGF1 promotes the proliferation of NIH/3T3 cells and significantly stimulates wound healing in the diabetic wounded rat model. Therefore, KGF1 is suggested as a prospective industrial production factor in plant bioreactors and a promising candidate for further development as a therapeutic agent for wound healing.

## References

[1] Takase HM, Itoh T, Ino S (2013). FGF7 is a functional niche signal required for stimulation of adult liver progenitor cells that support liver regeneration. *Genes & Development*.

[2] Kakizaki I, Itano N, Kimata K (2008). Up-regulation of hyaluronan synthase genes in cultured human epidermal keratinocytes by UVB irradiation. *Archives of Biochemistry and Biophysics*.

[3] Tandara AA, Kloeters O, Mogford JE, Mustoe TA (2007). Hydrated keratinocytes reduce collagen synthesis by fibroblasts via paracrine mechanisms. *Wound Repair and Regeneration*.

[4] Braun S, Mauch C, Boukamp P, Werner S (2007). Novel roles of NM23 proteins in skin homeostasis, repair and disease. *Oncogene*.

[5] Marti GP, Mohebi P, Liu L, Wang J, Miyashita T, Harmon JW (2008). KGF-1 for wound healing in animal models. *Methods in Molecular Biology*.

[6] Tsai SM, Wang WP (2011). Expression and function of fibroblast growth factor (FGF)7 during liver regeneration. *Cellular Physiology and Biochemistry*.

[7] Berent-Maoz B, Montecino-Rodriguez E, Signer RA, Dorshkind K (2012). Fibroblast growth factor-7 partially reverses murine thymocyte progenitor aging by repression of Ink4a. *Blood*.

[8] Santa Maria PL, Redmond SL, Atlas MD, Ghassemifar R (2011). Keratinocyte growth factor 1, fibroblast growth factor 2 and 10 in the healing tympanic membrane following perforation in rats. *Journal of Molecular Histology*.

[9] Raffa S, Leone L, Scrofani C, Monini S, Torrisi MR, Barbara M (2012). Cholesteatoma-associated fibroblasts modulate epithelial growth and differentiation through KGF/FGF7 secretion. *Histochemistry and Cell Biology*.

[10] Aguilar S, Scotton CJ, McNulty K (2009). Bone marrow stem cells expressing keratinocyte growth factor via an inducible lentivirus protects against bleomycin-induced pulmonary fibrosis. *PLoS ONE*.

[11] Mann K, Meng B (2013). The triple gene block movement proteins of a grape virus in the genus Foveavirus confer limited cell-to-cell spread of a mutant Potato virus X. *Virus Genes*.

[12] Kakareka NN, Volkov IuG, Kozlovskaia ZN, Pleshakova TI (2013). Production of immunodiagnosticum preparations based on plant virus strains. *Mikrobiolohichnyĭ Zhurnal*.

[13] Park MR, Kim KH (2013). Molecular characterization of the interaction between the N-terminal region of Potato virus X, (PVX) coat protein (CP) and Nicotiana benthamiana PVX CP-interacting protein, NbPCIP1. *Virus Genes*.

[14] Hefferon KL (2012). Plant virus expression vectors set the stage as production platforms for biopharmaceutical proteins. *Virology*.

[15] Tyulkina LG, Skurat EV, Frolova OY, Komarova TV, Karger EM, Atabekov IG (2011). New viral vector for superproduction of epitopes of vaccine proteins in plants. *Acta Naturae*.

[16] Wagner B, Fuchs H, Adhami F, Ma Y, Scheiner O, Breiteneder H (2004). Plant virus expression systems for transient production of recombinant allergens in Nicotiana benthamiana. *Methods*.

[17] Lacorte C, Ribeiro SG, Lohuis D, Goldbach R, Prins M (2010). Potato virus X and Tobacco mosaic virus-based vectors compatible with the Gateway cloning system. *Journal of Virological Methods*.

[18] Peng C, Chen B, Kao H-K, Murphy G, Orgill DP, Guo L (2011). Lack of FGF-7 further delays cutaneous wound healing in diabetic mice. *Plastic and Reconstructive Surgery*.

[19] Marti G, Ferguson M, Wang J (2004). Electroporative transfection with KGF-1 DNA improves wound healing in a diabetic mouse model. *Gene Therapy*.

